# Numerical modeling of the hydrodynamic behavior of MgO–water nanofluid in plate-fin heat exchangers under varying operating conditions

**DOI:** 10.1038/s41598-026-60276-5

**Published:** 2026-06-30

**Authors:** Abbas J. Sultan, Ali A. Yahya, Fadhl H. Faraj, Narinderjit Singh Sawaran Singh, Dheyaa J. Jasim, Laith S. Sabri, Haydar A. S. Aljaafari, Mahmut Taner, Ali Mohammadi Hasanabad

**Affiliations:** 1 College of Chemical Engineering, University of Technology- Iraq, Baghdad, 10066 Iraq; 2 Faculty of Data Science and Information Technology, INTI International University, Persiaran Perdana BBN, Putra Nilai, Nilai, 71800 Malaysia; 3 College of Engineering, University of Al Maarif, Al Anbar, 31001 Iraq; 4 Faculty of Engineering and Architecture, Istanbul Gelisim University, Istanbul, Turkey; 5 Fast Computing Center, Shabihsazan Ati Pars, Tehran, Iran

**Keywords:** Artificial neural network, Reynolds number, Pressure drop, Hydrodynamic properties, Flow rate, Industrial and innovation, Engineering, Mathematics and computing

## Abstract

This research examined the ability of an artificial neural network to predict flow behavior, defined by Reynolds number and pressure drop as functions of volume fraction and temperature, at fixed flow rates of 5, 8, and 11 LPM. The proposed framework was developed to address the nonlinear, interrelated thermo-compositional effects that influence flow inertia and pressure losses, without relying on predefined empirical relationships. The model’s reliability and predictive performance were evaluated through 5-fold cross-validation, monitoring of learning errors, regression diagnostics, error quantification, and sensitivity analysis. Results from cross-validation indicated stable Reynolds number predictions at low and moderate flow rates, with mean RMSE values of 6.39 at 5 LPM and 6.16 at 8 LPM. At 11 LPM, the mean RMSE rises to 13.87, highlighting the greater sensitivity and complexity of inertia-dominated flow regimes. Conversely, predictions of pressure drop remained consistently accurate across all operating conditions, achieving mean RMSE values of 2.68 mbar, 0.56 mbar, and 0.94 mbar at 5, 8, and 11 LPM, respectively. Regression analysis confirmed a strong linear relationship between predicted and reference values for both outputs, with correlation coefficients exceeding 0.99 at low and moderate flow rates and remaining above 0.96 at the highest flow rate. Further analysis of relative and absolute errors indicated high predictive accuracy, with mean relative errors below 5% for Reynolds number and below 1.3% for pressure drop across all scenarios. Sensitivity analysis consistently identified temperature as the primary source of predictive variability, with volume fraction being the secondary contributor. Pressure drop showed significantly lower sensitivity than Reynolds number, suggesting a smoother, less nonlinear relationship with the governing inputs.

## Introduction

Plate-fin heat exchangers (PFHEs) constitute a core class of compact HEs extensively employed in automotive thermal management systems, particularly in radiator applications where high heat dissipation rates must be achieved within strict geometric and weight constraints^1,2^. Owing to their extended heat transfer surface and compact configuration, PFHEs enable efficient thermal control; however, their practical performance is governed by a coupled interaction between thermal and hydrodynamic mechanisms within the finned flow passages^3,4^. From an operational standpoint, hydrodynamic behavior plays a decisive role in determining the overall efficiency of automotive cooling systems^5^. Pressure losses induced by complex fin geometries directly translate into increased pumping power requirements, thereby affecting system efficiency, fuel consumption, and long-term operational reliability^6^. Consequently, hydrodynamic parameters such as the Reynolds number (Re) and pressure drop (∆P) serve as fundamental performance indicators and must be rigorously characterized alongside thermal metrics during the design and optimization of PFHEs^7,8^.The sensitivity of these hydrodynamic characteristics is further amplified by variations in coolant properties and operating conditions^9^. Changes in inlet temperature (T), flow conditions, and fluid composition can significantly alter viscosity, density, and flow regime, leading to nonlinear, interdependent variations in Re and ∆P^10^. Such effects are particularly pronounced in compact HE, where small geometric scales intensify viscous effects and boundary-layer interactions. In this framework, the selection of advanced coolant formulations has emerged as a critical factor influencing PFHE performance. The introduction of nanoparticle (NP)-enhanced fluids modifies the effective thermophysical properties of the coolant and alters the momentum transport mechanisms within the flow channels^11,12^. While these modifications may enhance certain aspects of system performance, they also introduce additional complexity into the hydrodynamic response of the exchanger, making accurate prediction increasingly challenging^13^.Despite their widespread use, classical analytical approaches and empirical correlations are often developed under restrictive assumptions and exhibit limited capability to represent the nonlinear coupling between operating parameters and hydrodynamic responses in PFHEs, particularly when complex working fluids are employed^14,15^. Furthermore, comprehensive experimental characterization across broad operating ranges remains resource-intensive and impractical for iterative design and optimization processes. As a result, the accurate and efficient prediction of hydrodynamic behavior in PFHEs under varying operating conditions remains an open, technically significant challenge in modern automotive thermal system design^16^. Ozbalci et al.^17^ experimentally investigated the thermal performance of plate-fin and pin-fin heat sinks used in electronic cooling systems employing pure water and an Al₂O₃–water nanofluid with a φ of 0.1%.The results indicated that, for a smooth surface, the nanofluid enhanced heat transfer by up to 10.5% compared with the base fluid. The plate-fin heat sink achieved maximum heat transfer improvements of approximately 64.25% with water and 82.8% with the nanofluid, while the pin-fin heat sink provided corresponding enhancements of 56.4% and 70.27%.Elibol et al.^18^ numerically investigated the hydrodynamic and thermal performance of TiO₂–water nanofluid flowing through an offset strip fin channel in a heavy-vehicle radiator under three-dimensional laminar conditions. Their results showed that increasing Re and NP-φ enhanced heat transfer characteristics but significantly increased ∆P and pumping power due to higher viscosity. At low NP-φ, the nanofluid offered no notable advantage over pure water, whereas at higher φs, overall performance improved, with the performance evaluation criterion increasing by 7.2% at Re = 300 and 13% at Re = 800 when φ increased from 0 to 4%.Rajangam et al.^19^ numerically analyzed the thermal and hydrodynamic performance of an aluminum pin-fin heat sink using SiC–alkaline water and ZnO–alkaline water nanofluids at φs of 0.25–2% under a constant heat flux of 118.9 kW m⁻². The results indicated that NP addition improved heat transfer but increased flow resistance. At φ = 2%, pumping power rose by approximately 17% and 33%, respectively, while the average heat transfer coefficient increased by up to 18% and 16% at lower φs before decreasing by about 14% and 12% at higher loadings. Farooq et al.^20^ numerically investigated the thermal and hydrodynamic performance of plate-fin and pin-fin heat sinks using a GnP–MWCNT/water hybrid nanofluid over a range of Re and NP-φ. Their results indicated that the pin-fin configuration intensified flow disturbances and improved convective heat transfer, but at the expense of higher ∆P and thermal resistance. By contrast, the plate-fin heat sink exhibited lower flow resistance and achieved higher overall effectiveness. Quantitatively, at Re = 5334 and φ = 0.20%, the ∆P reached 88.5 Pa for the pin-fin heat sink compared with 73 Pa for the plate-fin design. Moreover, at Re = 1333, the effectiveness of the plate-fin heat sink increased from 0.333 for water to 0.354 for the nanofluid, remaining consistently higher than that of pin-fin configuration.Prediction of hydrodynamic behavior in nanofluid-based PFHEs was explored mainly through experimental measurements and numerical simulations; however, these approaches were often restricted to specific operating conditions and relied on empirical correlations that struggle to capture the nonlinear coupling between inlet T, NP-φ, and flow resistance in compact finned geometries. Such limitations reduced their flexibility and accuracy when extended beyond calibrated ranges. In recent years, data-driven techniques emerged as effective alternatives, offering the ability to learn complex nonlinear relationships directly from experimental data without imposing predefined functional forms. Building on this perspective, the present study introduced a machine-learning-based predictive framework to model the hydrodynamic behavior of a plate-fin heat exchanger operating with an MgO–water nanofluid. The model was formulated to predict Re and ∆P as output variables using inlet T and NP-φ as inputs, while the predictive performance was systematically examined under three distinct Flow rate (FR) of 5, 8, and 11 LPM. By adopting this strategy, the study provided a flexible and computationally efficient approach for hydrodynamic prediction that captured flow behavior across discrete flow regimes, thereby offering a practical tool for flow assessment and design analysis of nanofluid-cooled PFHEs.

## Train/test methodology

In the present study, a supervised artificial neural network (ANN) was employed as a data-driven modeling framework to predict the hydrodynamic behavior of a plate-fin heat exchanger operating with an MgO–water nanofluid^21^. The ANN was formulated using a feedforward architecture, in which information propagates unidirectionally from the input layer to the output layer through a hidden layer, without any recurrent or feedback connections^22^. This choice was appropriate for the present problem, since the hydrodynamic outputs depended solely on instantaneous operating conditions rather than on temporal or history-dependent effects^23,24^. The network was designed to approximate a nonlinear functional relationship between the governing operating parameters and the resulting hydrodynamic responses. Inlet T and NP-φ were selected as input variables, as they directly affected the nanofluid’s effective viscosity and density and therefore governed flow resistance within the finned channels, while Re and ∆P were defined as output variables representing the primary hydrodynamic indicators of the flow regime and pumping requirements. The experimental dataset employed for ANN development was obtained from the thermo-hydraulic investigation reported by Aktas et al.^25^, where an MgO–water nanofluid was experimentally examined in a plate-fin heat exchanger equipped with offset-strip fins on the coolant side and wavy fins on the air side. The investigated operating conditions included inlet temperatures ranging from 40 to 80 °C, nanoparticle volume fractions from 0 to 0.2%, and three discrete coolant flow rates of 5, 8, and 11 LPM. However, the original experimental study reported multiple thermo-hydraulic outputs; the present ANN framework specifically utilized Re and ∆P as the target hydrodynamic output parameters. Table 1.

**Table 1:** Summary of the experimental dataset.

Parameter	Value/range
NF	MgO-Water
NP size	18 nm
Inlet temperature	40–80 °C
φ	0–0.2%
Flow rates	5, 8, 11 LPM
Total samples	75
Outputs	Re, ΔP
PFHE configuration	Offset-strip/wavy fin

**Fig. 1:**
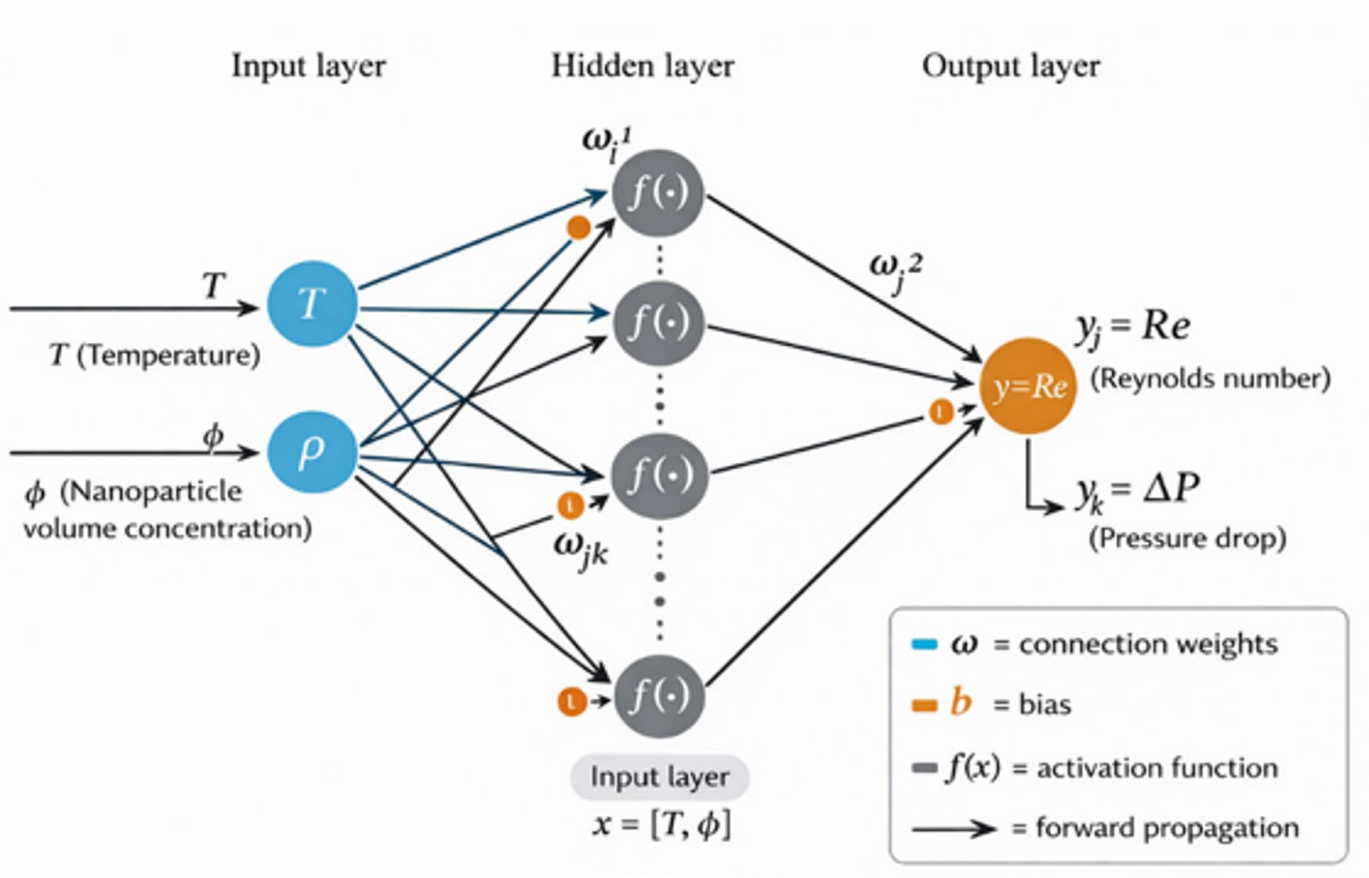
Architecture of a two-layer backpropagation neural network.

In the source experimental study, the measurement procedures, calibration conditions, and uncertainty analyses were systematically evaluated before data acquisition. The reported experimental uncertainties were approximately 4% for the heat transfer rate, 5.5% for the heat transfer coefficient, and 6% for the Nusselt number. Furthermore, all measurements were acquired after the experimental system stabilized under continuous operating conditions, thereby improving the reliability and consistency of the dataset used for ANN training and validation. ANN consisted of a single hidden layer with five neurons, a configuration selected after systematic testing of alternative architectures to ensure sufficient nonlinear approximation while maintaining model parsimony and avoiding overfitting, particularly critical given the limited size of the available dataset. A nonlinear activation function was applied in the hidden layer to capture the intrinsic nonlinear coupling between the input parameters and the hydrodynamic response, whereas a linear activation function was used in the output layer to preserve the continuous, physically meaningful nature of the predicted quantities. The experimental data were organized by operating flow rate, and for each FR (5, 8, and 11 LPM), an independent dataset of 25 samples was used. Separate ANN models were therefore trained for each flow-rate condition to preserve the physical distinction between the different operating regimes. Accordingly, the developed ANN models should be interpreted as condition-specific nonlinear predictive frameworks rather than as a unified global representation spanning all flow-rate regimes simultaneously. Within each dataset, the samples were randomly divided into 60% for training, 20% for validation, and 20% for testing. To determine an appropriate ANN architecture, several hidden-layer configurations containing 3–10 neurons were systematically evaluated. Since ANN training is sensitive to the initial distribution of weights and biases, each candidate architecture was trained repeatedly with different random initializations to reduce initialization-dependent bias and improve the robustness of model selection. The final network configuration, consisting of a single hidden layer with five neurons, was selected based on the minimum validation mean squared error (MSE) and stable predictive consistency across the training, validation, and test datasets, while avoiding unnecessary model complexity and overfitting. Importantly, the independent test subset was not involved in architecture selection or parameter tuning and was used only for the final evaluation of predictive performance. Before training, all input and output variables were normalized to the interval [0, 1] using the built-in mapminmax preprocessing function in the MATLAB ANN framework. The normalization mapping was established during training and consistently applied to the validation and test sets using the same scaling parameters, thereby minimizing the risk of information leakage and preserving the independence of unseen data. Model training was performed iteratively using the Levenberg–Marquardt (LM) optimization algorithm, during which the network weights and biases were adjusted to minimize the discrepancy between predicted and target values using the MSE criterion. The validation subset was continuously monitored throughout the learning process, and an early-stopping strategy was implemented by terminating training when no improvement in validation performance was observed for six consecutive validation checks (max_fail = 6). This procedure reduced the risk of overfitting while improving the generalization capability of the developed ANN framework for hydrodynamic prediction of nanofluid-based PFHEs under discrete operating conditions. The experimental measurements used for ANN development were acquired under stabilized operating conditions, approximately 30 min after continuous system operation began, as reported in the source experimental study. Figure 1.

### LM

In the present study, the ANN was trained using the LM optimization scheme, which was specifically well-suited for nonlinear regression problems with limited datasets. The LM algorithm was formulated to efficiently minimize the discrepancy between predicted and target values by dynamically adjusting the search direction during training^26,27^. Through this adaptive formulation, the algorithm transitions smoothly between gradient-based updates and curvature-informed corrections, enabling both stable and rapid convergence during weight optimization. The effectiveness of the LM algorithm was particularly pronounced in problems characterized by strong nonlinearity between inputs and outputs, such as the hydrodynamic response of nanofluid-based HEs. In contrast to conventional first-order optimization techniques, which may require extensive iterations or be sensitive to local minima, the LM approach provided improved robustness by regulating the update step via an adaptive damping parameter. This mechanism automatically balanced convergence speed and numerical stability throughout training.

1 $$\:f=\sum\:_{i=1}^{m}{e}_{i}^{2}$$

Within the adopted learning framework, the total number of available samples is denoted by m, which defines the dimensionality of the optimization problem. The training process relied on a sensitivity-based representation of the objective function, in which the network’s local response to parameter variations was systematically quantified. This information was embedded in a Jacobian matrix formed from the first-order derivatives of prediction residuals with respect to network weights. Such a formulation provided a mathematically consistent description of how perturbations in the parameter space affected the model response, thereby enabling efficient, well-conditioned updates to the network parameters during optimization. By incorporating this sensitivity structure, the learning process converges more quickly while preserving the network’s ability to represent the underlying nonlinear relationships between inputs and outputs.

2 $$\:{J}_{i,J}=\frac{\partial\:{e}_{i}}{\partial\:{W}_{i}}$$

In the considered formulation, the optimization problem is defined over a finite set of p model parameters. The learning process proceeds by evaluating the local variation of the objective function with respect to each adjustable parameter, thereby constructing a gradient vector in the parameter space. This gradient encapsulated the sensitivity of the prediction error to infinitesimal changes in the network parameters and served as the fundamental guide for parameter updates during training. By systematically accounting for each parameter’s contribution to the overall loss, the optimization algorithm iteratively steers the model toward a configuration that minimizes the discrepancy between predicted and reference values.

3 $$\:\nabla\:f=2{J}^{T}.e$$

In the proposed formulation, the vector of residuals aggregates the deviations between the network predictions and the corresponding target values over the entire training set. This residual information was exploited to construct an approximate representation of the second-order characteristics of the objective function, capturing the local curvature of the error landscape. By incorporating this curvature-related information into the optimization process, the learning algorithm can adjust network weights and biases more effectively. Such an approximation significantly improved convergence efficiency and numerical robustness, enabling stable training performance even when the available data were limited in size or subject to measurement uncertainty.

4 $$\:H\approx\:2{J}^{T}.J.\lambda.I.$$

Within the optimization scheme, a scalar control parameter was introduced to regulate the magnitude of parameter updates during training. This parameter played a critical role in balancing numerical robustness and convergence efficiency by modulating the influence of curvature information in the update rule. Increasing its value made the optimization process more conservative, leading to greater stability in regions where the error surface was ill-conditioned. Conversely, reducing this parameter allowed the algorithm to exploit second-order information more aggressively, thereby accelerating convergence toward the minimum of the objective function. Through this adaptive adjustment mechanism, the training process maintains both stability and efficiency across different stages of learning.

5 $$\:{W}^{(i+1)}={W}^{\left(i\right)}-{\left({J}^{\left(i\right)T}.{J}^{\left(i\right)}.{\lambda\:}^{\left(i\right)}I\right)}^{-1}.(2{J}^{i\left(T\right)}.{e}^{\left(i\right)})$$

### Performance indicators

The predictive capability of proposed ANN models was systematically evaluated using a set of complementary statistical indicators. These metrics were selected to provide a comprehensive assessment of model performance by examining both the magnitude of prediction errors and the agreement between model outputs and corresponding experimental observations. The mean squared error (MSE) and root mean square error (RMSE) quantify the dispersion of prediction residuals and thereby reflect overall accuracy, while the correlation coefficient (R) characterizes the strength of the linear association between predicted and measured values. Taken together, these performance measures offered an integrated evaluation of prediction accuracy, consistency, and reliability, with each metric highlighting a distinct aspect of the model’s predictive behavior.

#### The MSE

The MSE was adopted as a fundamental criterion for characterizing the predictive accuracy of the developed model. By defining the error as the average of squared deviations between predicted and measured values, this metric inherently emphasizes larger discrepancies and thus provides a rigorous assessment of the model’s fit^28^. From a modeling perspective, MSE showed the extent to which the learning process minimized residual errors across both training and test data, making it a key indicator of convergence behavior and numerical robustness. A reduction in MSE indicated improved alignment between model outputs and experimental observations, suggesting that the underlying nonlinear mapping was captured with greater fidelity. The mathematical definition of the MSE is given by:

6 $$\:MSE=\frac{1}{n}\sum\:_{i=1}^{n}{({P}_{oi}-{P}_{si})}^{2}$$

#### The RMSE

The RMSE was employed as a complementary performance metric to provide a physically interpretable measure of prediction accuracy. By expressing the error in the same units as the predicted variable, RMSE provides a direct measure of the typical magnitude of deviations between model outputs and experimental observations. This metric effectively captured residual dispersion while retaining sensitivity to larger errors, thereby bridging rigorous error penalization with practical interpretability. Consequently, RMSE served as a reliable indicator of the model’s overall predictive precision, with smaller values reflecting tighter clustering of predictions around the measured data and demonstrating the model’s ability to reproduce the underlying physical behavior faithfully. The mathematical expression of RMSE is defined as:

7 $$\:RMSE=\sqrt{\frac{1}{n}\times\:{\left[\sum\:_{i=1}^{n}({P}_{oi}-{P}_{si})\right]}^{2}}$$

#### The R

The R statistic was used to evaluate the degree of agreement between the model-predicted values and the corresponding experimental data. This metric quantified the strength of linear association between predictions and observations, providing insight into how effectively the model captured the data’s underlying trend, regardless of absolute error magnitude. Unlike error-based metrics, the R emphasizes consistency in prediction behavior and the preservation of physical trends across the dataset. Values of R approaching unity indicate a strong correspondence between predicted and measured values, confirming that the developed model accurately captured the systematic relationship governing the system’s hydrodynamic response. The mathematical definition of R is given by:

8 $$\:R=\sqrt{\frac{\sum\:_{i=1}^{n}{\left[\left({P}_{si}-{\stackrel{-}{P}}_{s}\right)\left({P}_{oi}-{\stackrel{-}{P}}_{o}\right)\right]}^{2}}{\sum\:_{i=1}^{n}{\left({P}_{si}-{\stackrel{-}{P}}_{s}\right)}^{2}\sum\:_{i=1}^{n}{\left({P}_{oi}-{\stackrel{-}{P}}_{o}\right)}^{2}}}$$

## Result and discussion

Owing to the limited size of the available experimental dataset, the developed ANN framework should be primarily interpreted as a condition-specific predictive interpolation model within the investigated operating domain rather than as a universally applicable predictive tool. To improve model robustness and reduce initialization-dependent bias, multiple randomized ANN initializations and hidden-layer configurations were systematically evaluated using independent validation and testing subsets. Furthermore, comparative analyses against regression-based baselines were conducted to assess whether the developed ANN captured meaningful nonlinear predictive behavior beyond simple linear interpolation.

### K-fold cross-validation

**Fig. 2:**
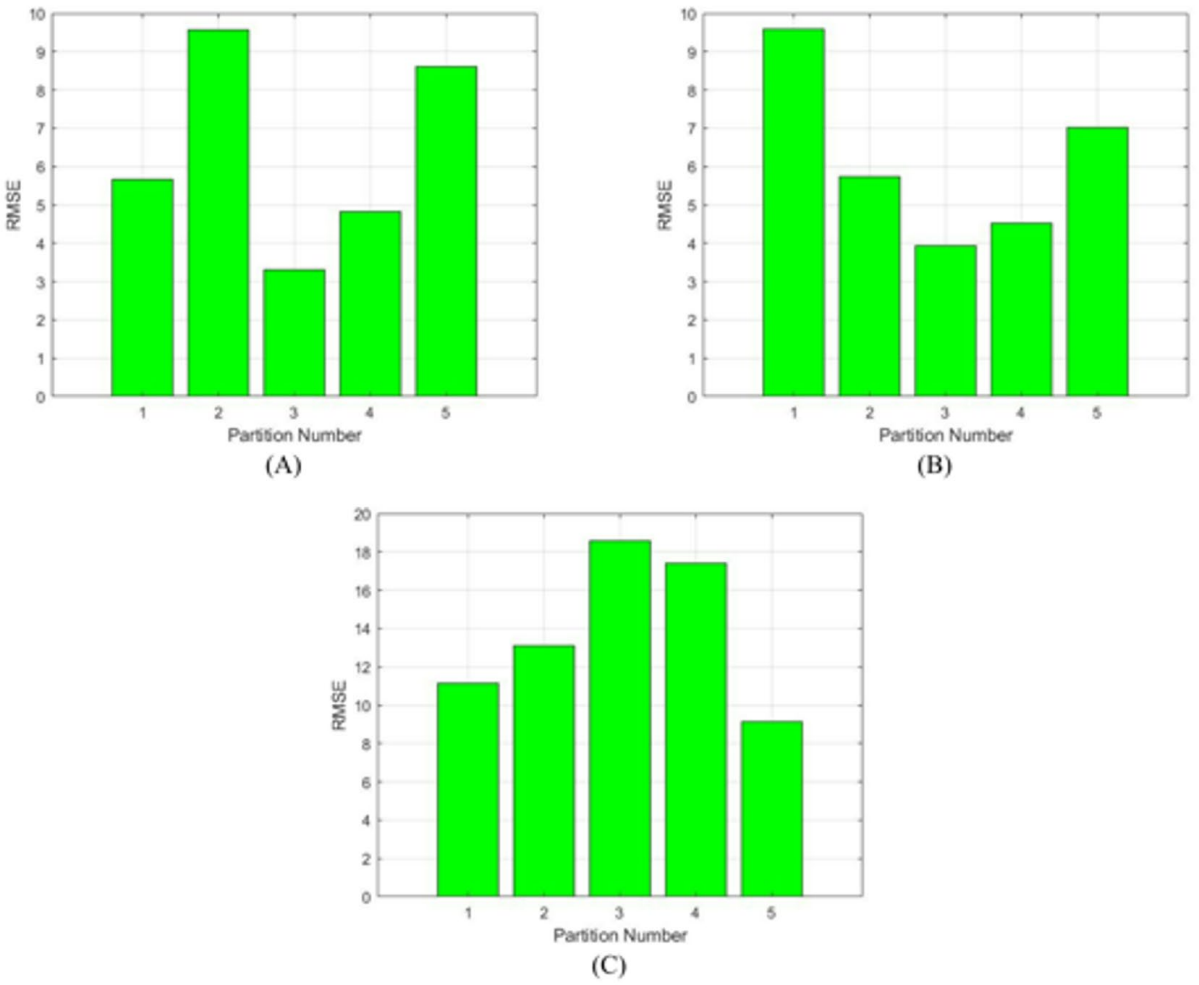
RMSE distributions of Re predictions obtained from 5-fold cross-validation analysis at FRs of (**A**) 5 LPM, (**B**) 8 LPM, and (**C**) 11 LPM.

To ensure a statistically robust and unbiased evaluation of the predictive capability of the developed model, a 5-fold cross-validation framework was adopted for all investigated operating conditions. For each FR, the dataset was randomly divided into five mutually exclusive subsets of comparable size. In each fold, four subsets were used for training, and the remaining subset served as the validation dataset, ensuring that each sample was evaluated exactly once. The predictive accuracy was quantified by averaging the error metrics across all folds, thereby minimizing sensitivity to data partitioning and improving the reliability of the performance assessment. Figure 2 presents RMSE distributions obtained from 5-fold cross-validation analysis for Re prediction at FRs of (A) 5 LPM, (B) 8 LPM, and (C) 11 LPM. At 5 LPM, the proposed ANN achieved an average MSE of 46.32 and a corresponding RMSE of 6.39, while the fold-wise RMSE values ranged from 3.30 to 9.56. The observed variation reflects localized differences in the hydrodynamic characteristics within individual validation subsets. At 8 LPM, the ANN maintained comparable prediction accuracy despite the increased sensitivity of Re to velocity variations and to effective hydraulic characteristics. Under this condition, the average MSE and RMSE reached 42.01 and 6.16, respectively, while the RMSE values varied between 3.94 and 9.59 across the five folds. The absence of excessive error amplification indicates that the learned input–output relationship remains stable under moderately inertia-dominated conditions. At 11 LPM, the predictive complexity increased noticeably due to intensified inertial effects and the enhanced sensitivity of Re to local flow variations. Consequently, the average MSE increased to 205.59, with a corresponding RMSE of 13.87, while the RMSE values ranged from 9.12 to 18.59. Although the error dispersion widened at this elevated FR, the RMSE values remained bounded, with no evidence of systematic instability or divergence.

**Fig. 3:**
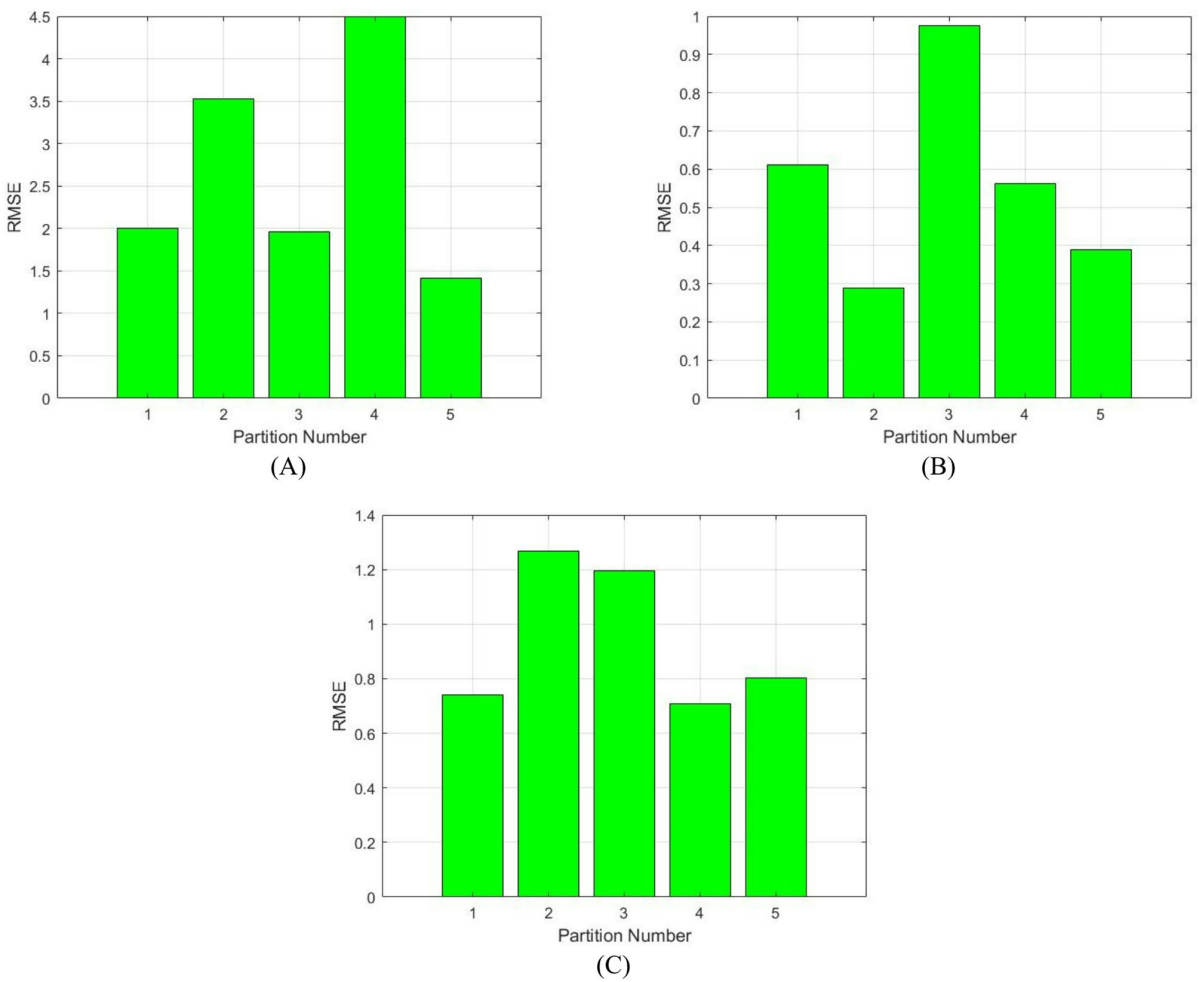
RMSE distributions of ∆P predictions obtained from 5-fold cross-validation analysis at FRs of (**A**) 5 LPM, (**B**) 8 LPM, and (**C**) 11 LPM.

Figure 3 illustrates the RMSE distributions obtained from 5-fold cross-validation analysis for ∆P prediction at FRs of (A) 5 LPM, (B) 8 LPM, and (C) 11 LPM. At 5 LPM, the ANN demonstrated strong predictive capability, yielding an average MSE of 8.51 mbar² and a corresponding RMSE of 2.68 mbar. The fold-wise RMSE values ranged from 1.41 to 4.50 mbar, indicating a relatively narrow, well-bounded distribution of prediction errors across the investigated validation subsets. At 8 LPM, the predictive accuracy improved further, with an average MSE of 0.37 mbar² and a corresponding RMSE of 0.56 mbar. Under this condition, the RMSE values ranged from 0.29 to 0.98 mbar, confirming that the ANN maintained stable predictive performance despite the increasing influence of viscous dissipation and inertial effects at elevated FRs. At 11 LPM, the average MSE and RMSE reached 0.95 mbar² and 0.94 mbar, respectively, while the RMSE values varied from 0.71 to 1.27 mbar across the five folds. Although the flow regime became increasingly inertia-dominated at this operating condition, the limited RMSE dispersion indicates that the ANN maintained stable, reliable predictive behavior without abrupt error escalation. Compared with Re prediction, the consistently lower RMSE values for ∆P across all FRs indicate a smoother, less sensitive dependence of ∆P on the governing operating parameters.

## Prediction results and analysis

### Comparing various performance functions

**Fig. 4:**
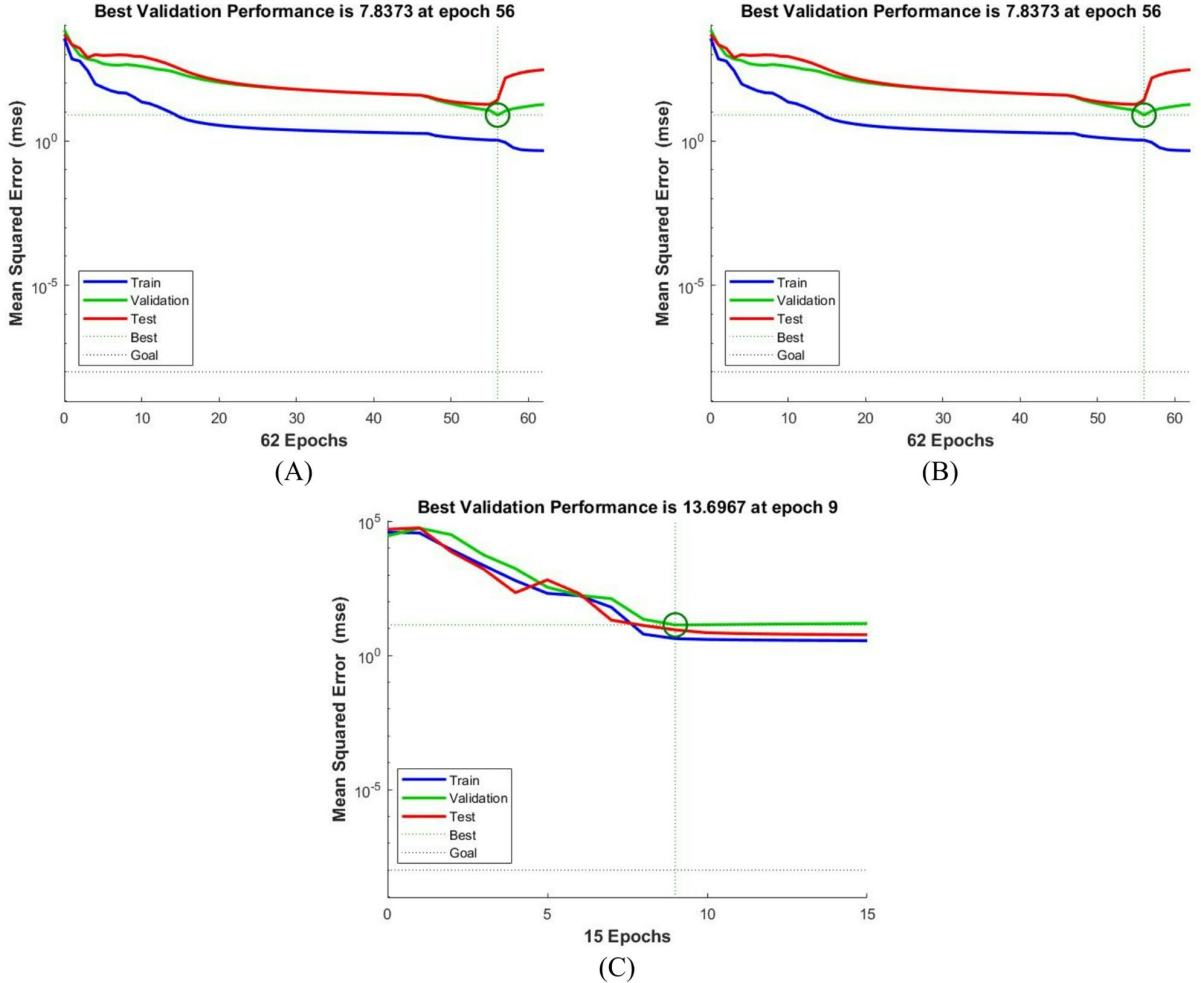
ANN performance diagrams based on MSE evolution for Re prediction during the training, validation, and testing stages at FRs of (**A**) 5 LPM, (**B**) 8 LPM, and (**C**) 11 LPM.

Figure 4 presents the ANN performance diagrams based on the MSE evolution for Re prediction during the training, validation, and testing stages at FRs of (A) 5 LPM, (B) 8 LPM, and (C) 11 LPM. At 5 LPM, the distribution of MSE values provided insight into the fidelity with which the model captured the governing hydrodynamic behavior underlying Re prediction. The low training MSE of 1.07 indicated that the dominant flow relationships embedded within the calibration dataset were effectively represented in the learned parameter space. The validation MSE of 7.84, which remained within the same order of magnitude as the overall MSE of 7.41, demonstrates that the learned input–output relationship maintained acceptable predictive consistency across statistically distinct operating states. In contrast, the higher test MSE of 26.00 indicated the presence of localized flow conditions characterized by enhanced variability in effective hydraulic behavior and velocity distribution, thereby naturally increasing prediction uncertainty. At 8 LPM, the MSE distribution showed the increasing influence of inertial effects on Re prediction. Under this condition, the training MSE reached 4.22, indicating that the ANN maintained acceptable predictive accuracy despite the increasing complexity of the flow regime. The validation MSE increased to 13.70, suggesting the presence of flow states associated with enhanced sensitivity to local velocity fluctuations and nonlinear hydraulic responses. Such behavior was expected at elevated FRs, where small perturbations in the governing parameters can induce amplified variations in Re. The test MSE of 8.98 remained close to the overall MSE of 7.07, confirming that the increase in prediction error was localized rather than indicative of global model bias or overfitting. At 11 LPM, the MSE distribution showed increasing complexity in Re prediction under strongly inertia-dominated flow conditions. The training MSE increased substantially to 28.39, reflecting the intensified sensitivity of Re to local flow variations and velocity gradients at this operating condition. The validation MSE further increased to 86.20, indicating that certain validation subsets contain highly nonlinear flow states in which small deviations in effective hydraulic parameters induce disproportionately large changes in Re. Despite this increase in validation error, the test MSE of 40.62 remained close to the overall MSE of 42.40, confirming that ANN did not exhibit systematic instability when applied to independent datasets.

**Fig. 5:**
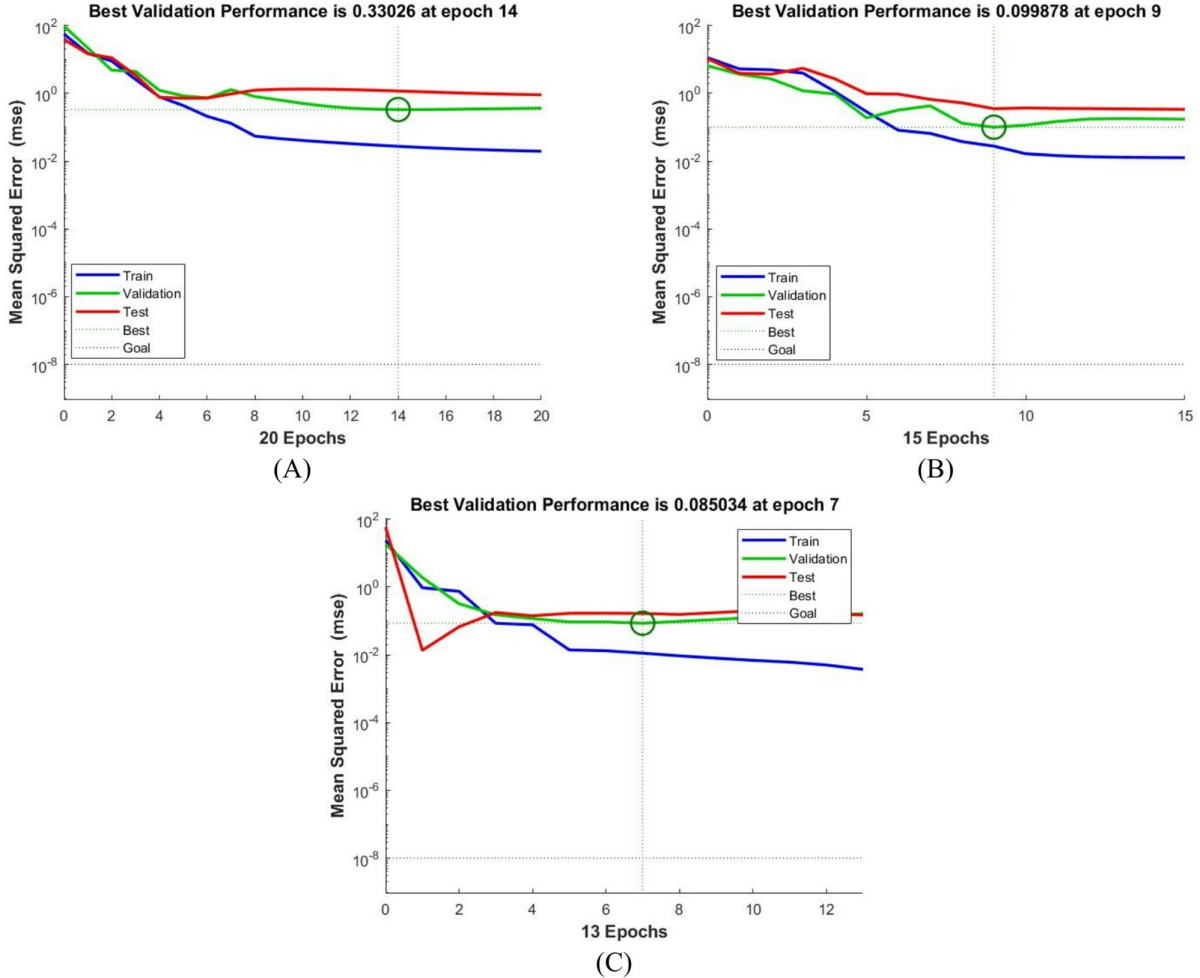
ANN performance diagrams based on MSE evolution for ∆P prediction during the training, validation, and testing stages at FRs of (**A**) 5 LPM, (**B**) 8 LPM, and (**C**) 11 LPM.

Figure 5 illustrates the ANN performance diagrams based on the MSE evolution for ∆P prediction during the training, validation, and testing stages at FRs of (A) 5 LPM, (B) 8 LPM, and (C) 11 LPM. At 5 LPM, a very low training MSE of 0.027 mbar² indicated that the ANN framework accurately captured the dominant viscous and wall-induced resistance mechanisms embedded within the calibration data. The validation MSE of 0.33 mbar² remained close to the overall MSE of 0.32 mbar², demonstrating that the learned relationship between the governing input variables and ∆P remained stable when evaluated using unseen operating conditions. The higher test MSE of 1.17 mbar² can be attributed to localized flow states associated with enhanced pressure gradients and increased sensitivity to frictional variations. At 8 LPM, the MSE distribution demonstrated that the ANN maintained stable predictive capability despite the increasing influence of flow inertia and viscous dissipation on ∆P behavior. The training MSE remained very low at 0.0276 mbar², indicating an accurate representation of the dominant hydraulic resistance mechanisms. The validation MSE of 0.0999 mbar² indicated that the learned mapping maintained stable predictive performance across statistically distinct operating states. The test MSE increased moderately to 0.3508 mbar², reflecting the increasing sensitivity of ∆P to localized pressure gradients and hydraulic nonuniformities at elevated FRs. However, the overall MSE of 0.1067 mbar² remained low, confirming that the developed ANN framework preserved robust predictive capability and acceptable generalization performance without evidence of systematic predictive deterioration. At 11 LPM, the MSE distribution showed the ANN’s ability to represent ∆P behavior under strongly inertia-dominated flow conditions. The training MSE of 0.0111 mbar² indicated that the dominant viscous and frictional effects governing ∆P remain accurately represented within the learned parameter space, even at elevated flow velocities. The validation MSE increased to 0.0850 mbar², suggesting the presence of operating states with enhanced sensitivity to local hydraulic resistance and intensified flow–wall interactions. The test MSE of 0.1643 mbar² remained within the same order of magnitude as the overall MSE of 0.0565 mbar², indicating that predictive performance remains stable for independent datasets. Overall, the limited error magnitudes across all data partitions confirmed that ∆P showed a smoother, less sensitive dependence on the governing operating parameters than Re, enabling the ANN to maintain stable, reliable predictive performance across the investigated FR range.

**Fig. 6:**
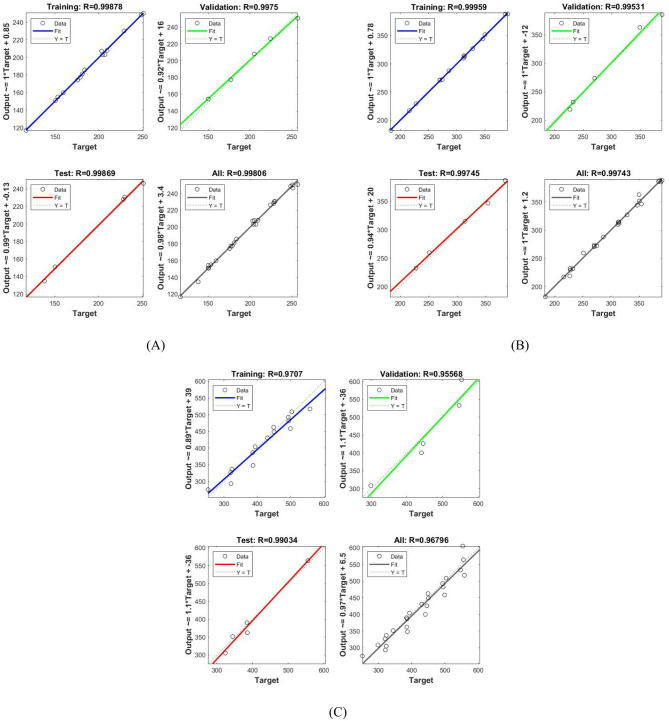
ANN regression diagrams for Re prediction at FRs of (**A**) 5 LPM, (**B**) 8 LPM, and (**C**) 11 LPM, including training, validation, testing, and overall datasets.

Figure 6 presents the ANN regression diagrams for Re prediction at FRs of (A) 5 LPM, (B) 8 LPM, and (C) 11 LPM, including the training, validation, testing, and overall datasets. At 5 LPM, the regression showed excellent linear correspondence between the predicted and target Re values. The obtained R values were 0.99878 for the training dataset, 0.99750 for the validation dataset, and 0.99869 for the test dataset, yielding an overall R value of 0.99806. The close clustering of data points around the 45° identity line indicated that ANN accurately captured the nonlinear relationship governing Re behavior under low-flow conditions. Moreover, the close agreement among the training, validation, and test correlations confirmed that the developed ANN maintained stable generalization without evidence of overfitting or systematic predictive degradation. At 8 LPM, the regression analysis indicated that ANN maintained strong predictive consistency despite the increasing influence of inertial effects on flow behavior. The obtained R values were 0.99959 for the training dataset, 0.99531 for the validation dataset, and 0.99745 for the testing dataset, yielding an overall R value of 0.99743. The consistently high correlation coefficients demonstrated that ANN preserved accurate mapping between the governing operating parameters and Re under moderately inertia-dominated conditions. The proximity of predicted values to the identity line further confirmed that the learned input–output relationship remained stable when evaluated using independent datasets. At 11 LPM, the regression behavior indicated increasing complexity in Re prediction under strongly inertia-dominated flow conditions. The obtained R values reached 0.97070 for the training dataset, 0.95568 for the validation dataset, and 0.99034 for the testing dataset, while the overall correlation coefficient remained high at 0.96796. Although the validation correlation decreased moderately relative to lower FRs, the obtained R values indicated a strong linear relationship between predicted and target Re values. The reduction in correlation can be attributed to Re’s heightened sensitivity to local velocity gradients and flow nonuniformities at elevated FRs, where small perturbations in hydraulic conditions can induce amplified variations in the dimensionless parameter.

**Fig. 7:**
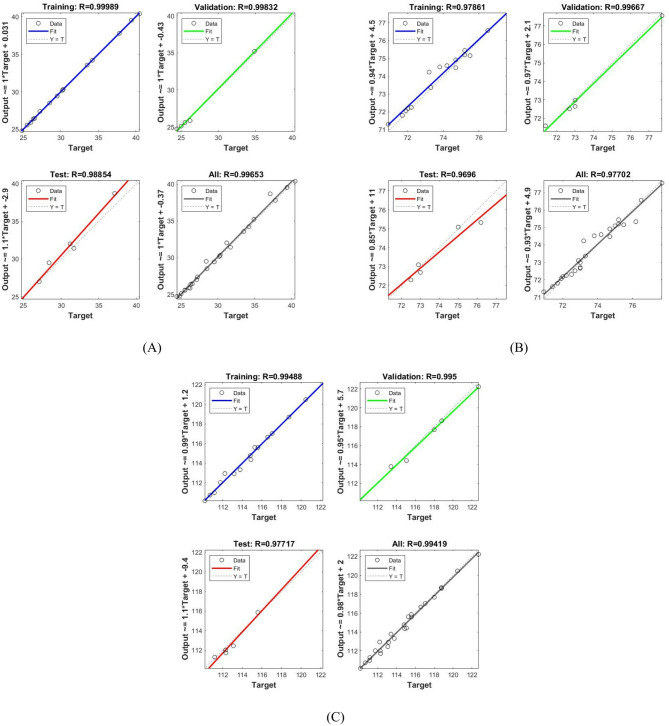
ANN regression diagrams for ∆P prediction at FRs of (**A**) 5 LPM, (**B**) 8 LPM, and (**C**) 11 LPM, including training, validation, testing, and overall datasets.

Figure 7 illustrates the ANN regression diagrams for ∆P prediction at FRs of (A) 5 LPM, (B) 8 LPM, and (C) 11 LPM, including the training, validation, testing, and overall datasets. At 5 LPM, the regression analysis demonstrated excellent agreement between the predicted and target ∆P values. The obtained R values were 0.99989 for the training dataset, 0.99832 for the validation dataset, and 0.98854 for the testing dataset, with an overall correlation coefficient of 0.99653. The strong clustering of predicted data around the identity line confirmed that the ANN successfully captured the dominant hydraulic resistance and viscous dissipation mechanisms governing ∆P behavior under low-flow conditions. At 8 LPM, the regression structure confirmed that the ANN preserved stable predictive capability under increasingly inertia-influenced operating conditions. The obtained R values were 0.97861 for the training dataset, 0.99667 for the validation dataset, and 0.96960 for the testing dataset, yielding an overall R value of 0.97702. Despite the moderate reduction in correlation relative to lower FRs, the overall regression behavior indicated that the dominant ∆P mechanisms remained accurately represented within the learned parameter space. The lower testing correlation can be attributed to the increasing sensitivity of ∆P to localized hydraulic resistance and flow nonuniformities at elevated FRs. At 11 LPM, the regression analysis showed that the ANN maintained high predictive reliability for ∆P estimation despite the increasing dominance of inertial effects and intensified momentum dissipation. The obtained R values reached 0.99488 for the training dataset, 0.99500 for the validation dataset, and 0.97717 for the testing dataset, while the overall correlation coefficient remained high at 0.99419. The preserved correlation structure across all data partitions indicates that the ANN consistently captured the dominant mechanisms governing ∆P behavior under high-flow conditions. Although the testing correlation decreased moderately compared with the training and validation datasets, the obtained R value indicated strong predictive capability.

**Fig. 8:**
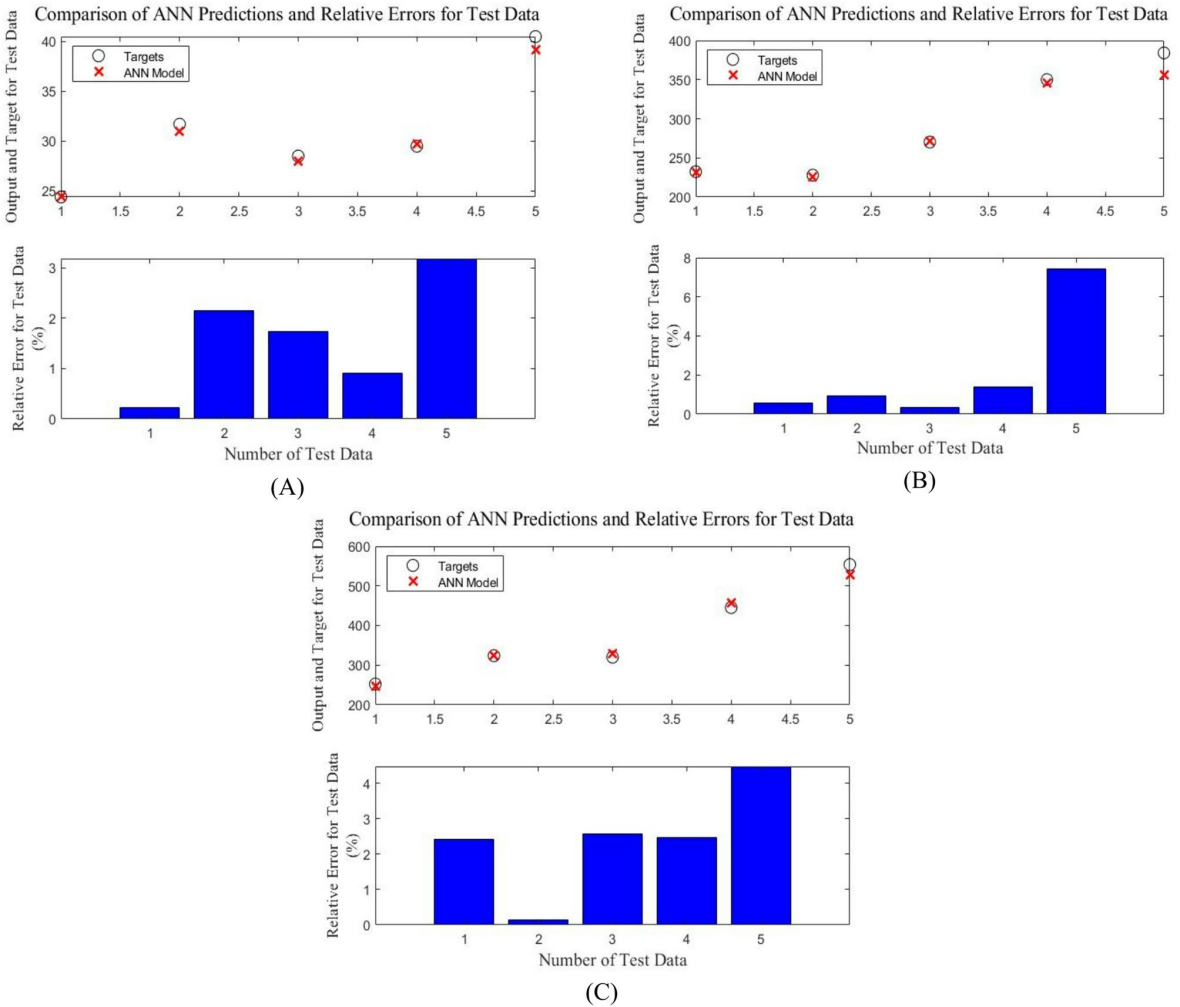
Correspondence plots and relative error distributions between Re values and ANN predictions for the test datasets at FRs of (**A**) 5 LPM, (**B**) 8 LPM, and (**C**) 11 LPM.

Figure 8 presents the correspondence plots and relative error distributions between the reference and predicted Re values for the test datasets at FRs of (A) 5 LPM, (B) 8 LPM, and (C) 11 LPM, where φ and T were employed as the governing input parameters. At 5 LPM, the predicted Re values closely match the reference data, confirming that the ANN effectively captures the coupled influence of thermal conditions and compositional variations on flow behavior. The relative error remained well controlled throughout the investigated operating range, varying from 0.85% to 3.21%, indicating a consistently high level of predictive fidelity. The limited spread of relative errors demonstrates that the ANN successfully captured the nonlinear dependence of Re on T-dependent fluid-property variations and φ-induced changes in flow characteristics without exhibiting localized predictive instability. At 8 LPM, predicted Re values continued to follow the reference trend with strong consistency, indicating that the ANN preserved acceptable predictive capability under increasingly inertia-dominated flow conditions. Under this operating condition, the relative error ranged from 0.35% to 7.42%. The moderate increase in the upper relative-error bound compared with lower FR conditions can be attributed to the enhanced sensitivity of Re to velocity fluctuations and T-dependent property variations at elevated FRs. In such regimes, small perturbations in φ or T may amplify variations in effective hydraulic behavior, thereby increasing the likelihood of localized prediction errors.

Nevertheless, the relative-error distribution remained bounded across the entire test range, confirming that the learned input–output mapping preserved stable predictive behavior without systematic loss of accuracy. At 11 LPM, the ANN continued to preserve strong agreement between the predicted and reference Re values despite the increasing complexity of the flow regime. The relative error remained within a bounded range of 0.15%–4.48%, confirming acceptable predictive reliability under highly inertia-dominated operating conditions. At elevated FRs, the influence of T-dependent fluid properties and φ-induced flow variations became increasingly pronounced because of intensified inertial effects and enhanced sensitivity to local hydrodynamic fluctuations.

**Fig. 9:**
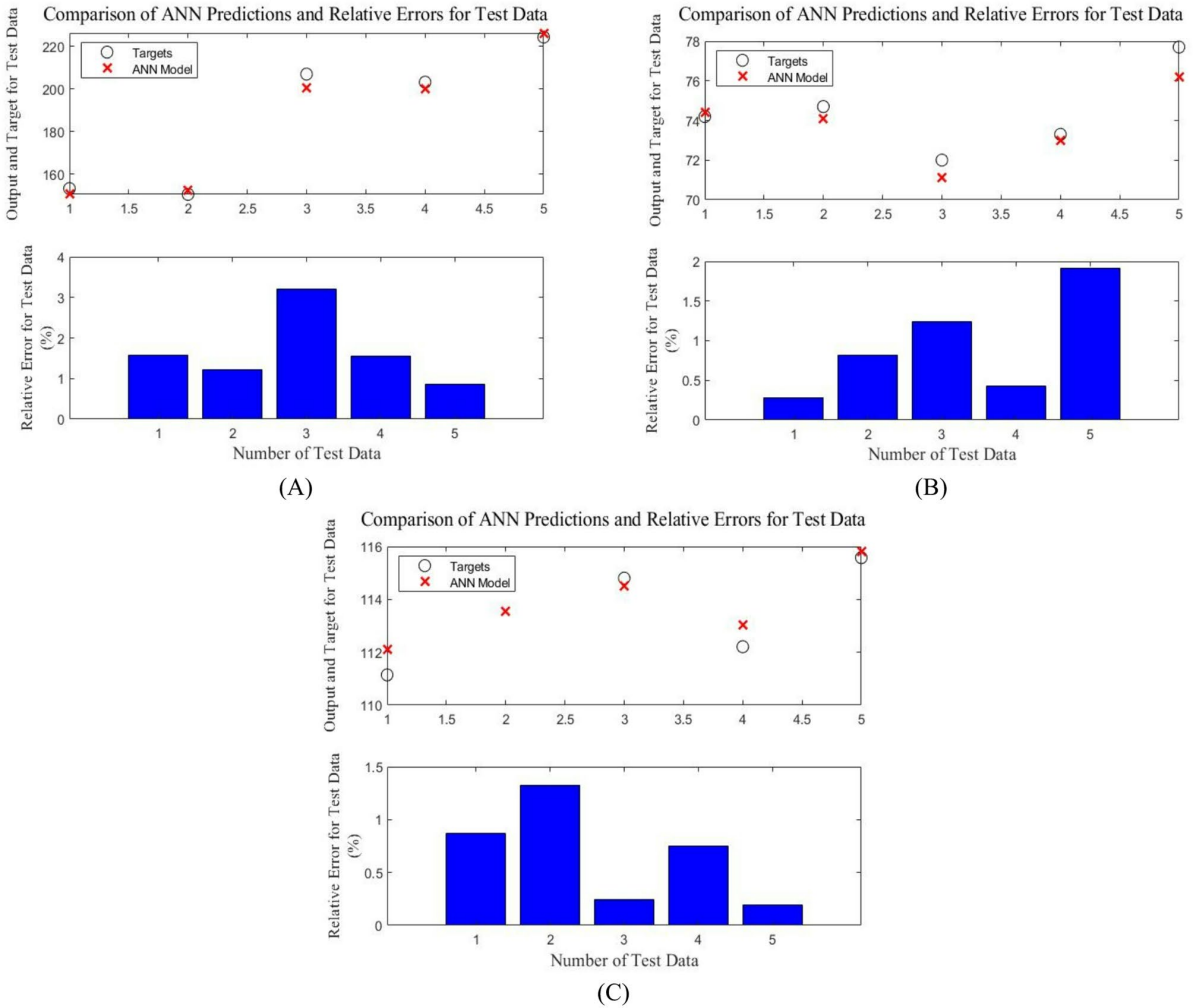
Correspondence plots and relative error distributions between ∆P values and ANN predictions for the test datasets at FRs of (**A**) 5 LPM, (**B**) 8 LPM, and (**C**) 11 LPM.

Figure 9 illustrates the correspondence plots and relative error distributions between the reference and predicted ∆P values for the test datasets at FRs of (A) 5 LPM, (B) 8 LPM, and (C) 11 LPM, where φ and T were used as the governing input variables. At 5 LPM, the predicted ∆P values closely followed the reference data, indicating that the ANN successfully captured the coupled thermal and compositional effects governing ∆P behavior. The relative error remained tightly bound across the investigated range, ranging from 0.22% to 3.18%, confirming a high level of predictive accuracy. The narrow relative-error distribution indicated that the nonlinear relationship between ∆P and the governing operating parameters was effectively represented within the learned parameter space. At 8 LPM, the ANN maintained strong agreement between the predicted and reference ∆P values, with a relative error within a narrow range of 0.28%–1.92%. Compared with the corresponding Re prediction at the same FR, the lower maximum relative error demonstrated that ∆P exhibits a smoother response to thermal and compositional variations even as inertial effects became increasingly important. The limited error spread confirmed that the ANN effectively captured the nonlinear dependence of ∆P on T-dependent fluid properties and φ-induced hydraulic resistance, without evidence of predictive instability. At 11 LPM, the predicted ∆P values continued to exhibit close correspondence with the reference data despite the increasing dominance of inertial effects and intensified momentum dissipation within the flow domain. The relative error remained tightly constrained between 0.19% and 1.33%, confirming consistently high predictive accuracy across the investigated operating range. Under these conditions, ∆P remained governed by the combined influence of viscous dissipation, hydraulic resistance, and T-dependent property variations, while preserving a comparatively smoother response to local flow perturbations than Re. Consequently, the ANN maintained a narrow relative-error distribution even at elevated FRs, demonstrating stable and reliable predictive behavior throughout the investigated operating conditions.

**Fig. 10:**
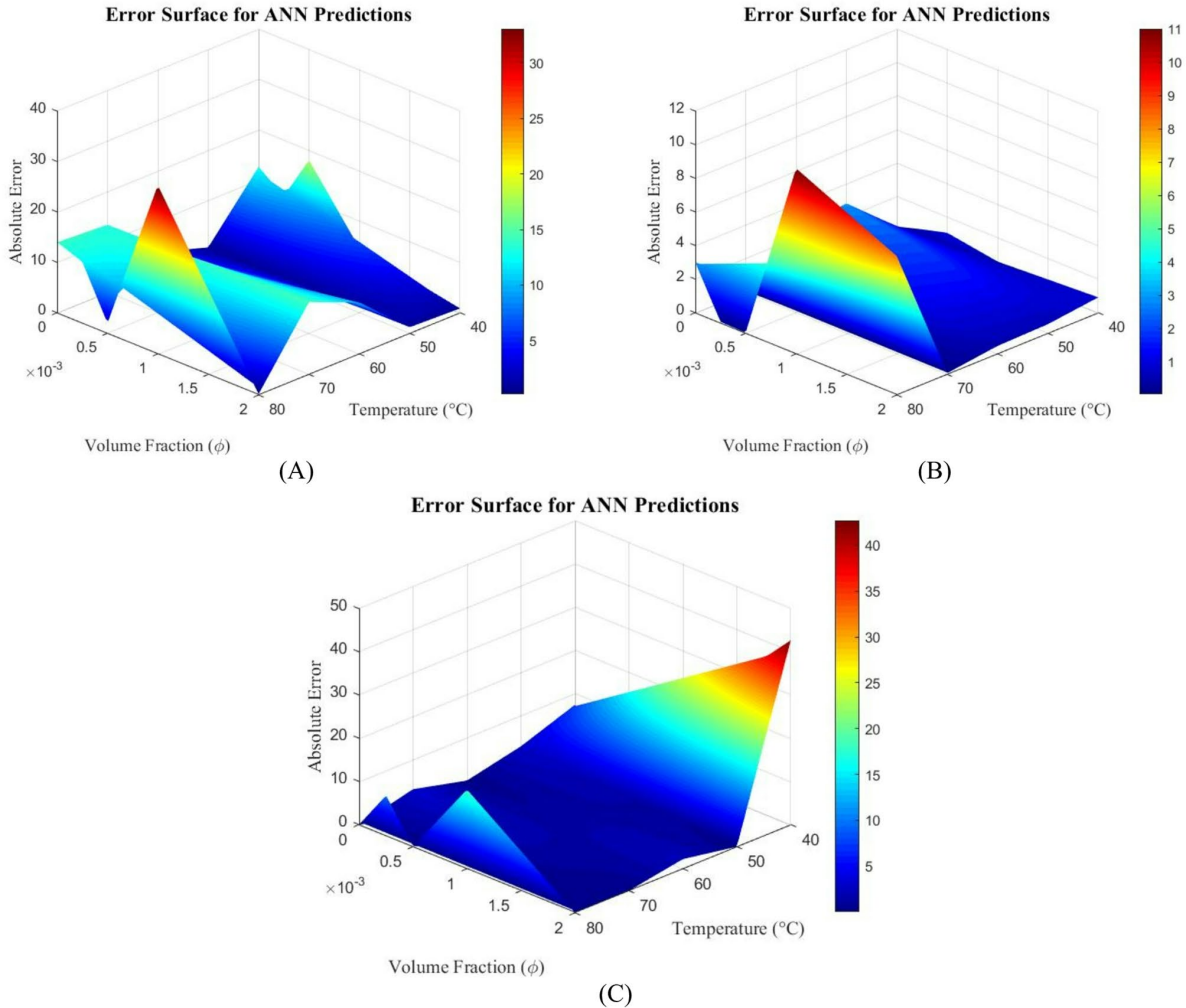
Distributions of absolute and relative prediction errors for Re estimation using the ANN model at FRs of (**A**) 5 LPM, (**B**) 8 LPM, and (**C**) 11 LPM.

Figure 10 presents the distributions of absolute and relative prediction errors for Re estimation using the developed ANN model at FRs of (A) 5 LPM, (B) 8 LPM, and (C) 11 LPM. At 5 LPM, the absolute prediction error remained well controlled across the investigated operating range, varying from 0.189 to 33.46, while the mean absolute error reached 8.83. These results indicate that the ANN successfully reproduced Re variations with limited deviation despite the simultaneous influence of thermal and compositional effects on flow behavior. In addition, the mean relative error remained low at 4.64%, confirming that prediction discrepancies remained small relative to the characteristic magnitude of Re under the investigated operating conditions. At 8 LPM, the error structure demonstrated that the ANN preserved strong predictive capability under increasingly inertia-dominated flow conditions. The absolute error remained confined within a relatively narrow range, varying from 0.034 to 11.05, while the mean absolute error reached 1.63. Compared with the corresponding results at 5 LPM, the reduced mean absolute error indicated improved consistency in the representation of Re behavior under moderately elevated FR conditions. Moreover, the mean relative error remained very low at 0.51%, confirming that prediction deviations remain limited relative to the characteristic Re magnitude. At 11 LPM, the prediction-error structure indicated increasing complexity in Re estimation under strongly inertia-dominated flow regimes. The minimum absolute error decreased to 7.95 × 10⁻⁶, indicating that the ANN achieved near-exact prediction accuracy for operating states characterized by well-defined flow trends. In contrast, the maximum absolute error increased to 42.71, while the mean absolute error reached 4.75, reflecting Re’s heightened sensitivity to localized variations in velocity distribution and effective transport behavior at elevated FRs. Nevertheless, the mean relative error remained limited to 1.29%, confirming that the normalized prediction deviations remain acceptably small relative to the characteristic Re magnitude.

**Fig. 11:**
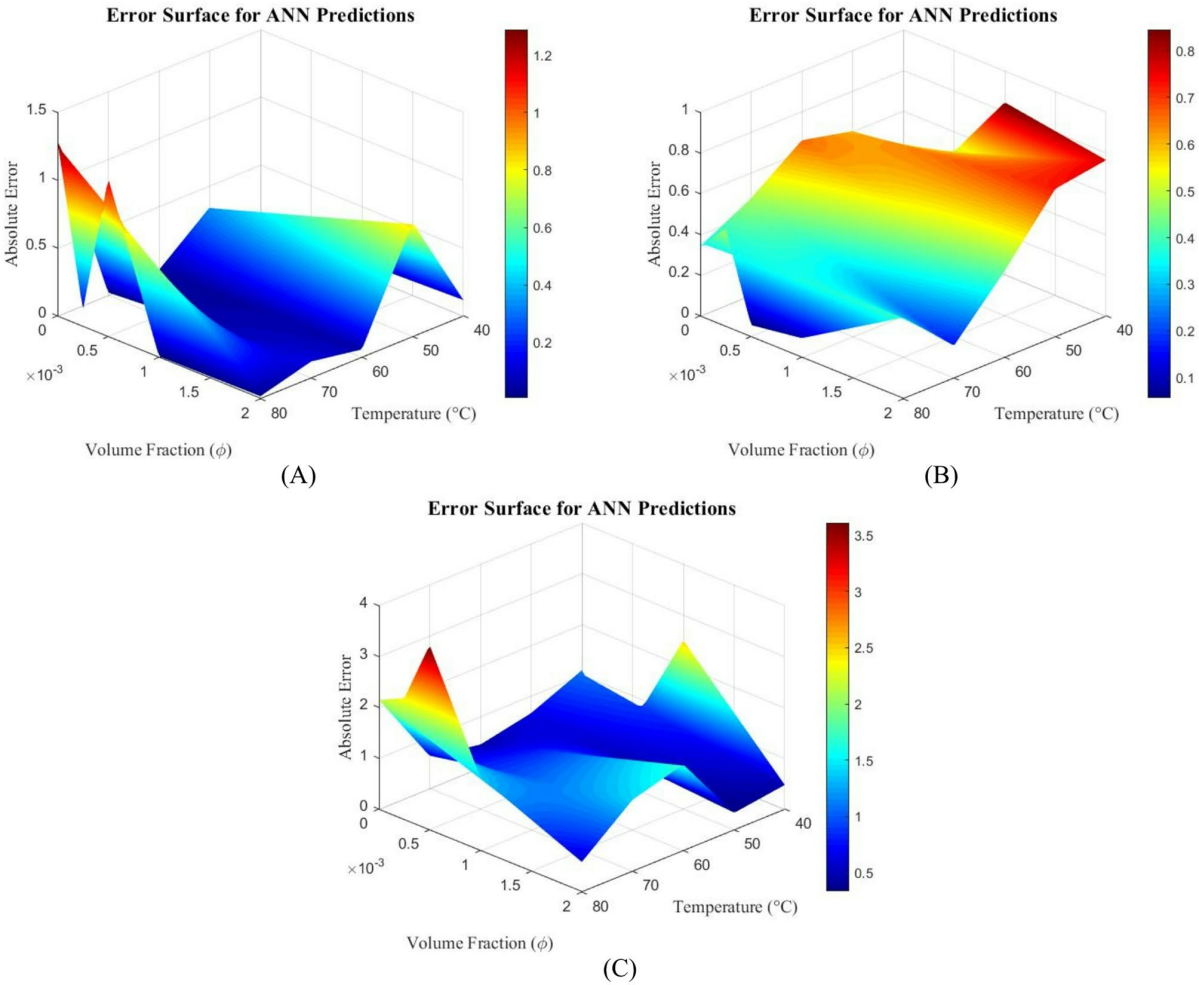
Distributions of absolute and relative prediction errors for ∆P estimation using the ANN model at FRs of (**A**) 5 LPM, (**B**) 8 LPM, and (**C**) 11 LPM.

Figure 11 illustrates the distributions of absolute and relative prediction errors for ∆P estimation using the developed ANN model at FRs of (a) 5 LPM, (b) 8 LPM, and (c) 11 LPM. At 5 LPM, the absolute error remained within a narrow range extending from 0.007 to 1.29 mbar, while the mean absolute error reached 0.28 mbar. The corresponding mean relative error remained low at 0.92%, indicating that the ANN accurately captured the dominant ∆P mechanisms, namely viscous dissipation and wall-induced flow resistance. At 8 LPM, the ANN maintained a similarly stable predictive behavior despite the increasing influence of inertial effects on ∆P. The absolute error varied between 0.056 and 1.06 mbar, while the mean absolute error reached 0.56 mbar. In addition, the mean relative error remained low at 0.77%, confirming that the ANN maintained accurate prediction capability even under elevated FR conditions. Compared with Re prediction at the same FR, the smaller relative-error magnitude indicated that ∆P showed a smoother response to thermal and compositional variations, thereby limiting localized error amplification and preserving stable predictive consistency. At 11 LPM, the error structure showed that the ANN maintained reliable ∆P prediction capability under strongly inertia-dominated flow conditions. The minimum absolute error reached 0.23 mbar, while the maximum absolute error increased to 4.01 mbar, and the mean absolute error reached 1.44 mbar. The increase in absolute error indicated greater sensitivity of ∆P to localized hydraulic resistance and intensified momentum dissipation at elevated FRs. Nevertheless, the corresponding mean relative error remained limited to 1.25%, indicating that the normalized prediction deviations remain small relative to the characteristic magnitude of ∆P.

#### Comparison with linear regression baseline

To further evaluate the predictive performance of the developed ANN framework, an additional comparison was conducted against a linear regression baseline model under identical training and test conditions. The comparative results indicated that the ANN generally provided superior predictive performance for Reynolds number estimation across the investigated flow-rate conditions, particularly at 5 and 8 LPM, where noticeable reductions in MSE and RMSE were achieved relative to the linear regression model. In contrast, pressure-drop prediction exhibited a more mixed behavior. At lower and moderate flow rates, the linear regression model demonstrated competitive or slightly improved prediction accuracy, suggesting that the ∆P response within restricted operating ranges partially follows smoother quasi-linear trends. However, at 11 LPM, the ANN again demonstrated improved predictive capability for ∆P estimation. These observations indicate that the ANN’s predictive advantage becomes more pronounced for hydrodynamic responses characterized by stronger nonlinear coupling with the governing operating parameters. Table 2.

**Table 2:** Comparative evaluation of the predictive performance of the developed ANN framework and the linear regression baseline for Re and ∆P prediction under different FRs.

FR		Model	MSE	RMSE	*R*
MLP5	Re	ANN	56.329	7.5053	0.991
		Linear	232.58	15.251	0.933
	∆P	ANN	7.89	2.81	0.848
		Linear	5.223	2.285	0.921
MLP8	Re	ANN	16.04	4.005	0.999
		Linear	711.96	26.68	0.908
	∆P	ANN	1.616	1.271	0.825
		Linear	0.156	0.395	0.998
MLP11	Re	ANN	856.52	29.266	0.960
		Linear	948.98	30.806	0.944
	∆P	ANN	0.257	0.507.	0.992
		Linear	0.874	0.934	0.963

### Sensitivity analysis

The sensitivity analysis at FR = 5 LPM indicated that T variations predominantly influenced the ANN response, whereas φ had a comparatively weaker influence on both output variables. As summarized in Table 3, increasing T perturbation from 2% to 10% increased the maximum variation of Re from 0.346% to 9.466%, while the corresponding variation induced by φ increased only from 0.078% to 0.572%. A similar trend was observed for the mean response values, where the mean Re variation increased from 0.212% to 2.447% under T perturbations, whereas φ perturbations produced a smaller increase from 0.027% to 0.171%. The sensitivity hierarchy is also preserved for ∆P prediction. Increasing T perturbation from 2% to 10% increased the maximum ∆P variation from 1.654% to 9.55%, while the corresponding φ-induced variation remained limited between 0.232% and 1.157%. Similarly, the mean ∆P variation increased from 0.71% to 4.07% under T perturbations, whereas φ variations produced a comparatively smaller increase from 0.062% to 0.310%. These results demonstrate that T-dependent variations exerted a substantially stronger influence on the ANN-predicted responses than equivalent perturbations in φ throughout the investigated operating range.

**Table 3:** Sensitivity of ANN-predicted Re and ∆P to variations in φ and T at 5 LPM.

ANN Output	Max change percentage in T					Max change percentage in φ				
	2	4	6	8	10	2	4	6	8	10
Re	0.346	1.732	4.62	7.739	9.466	0.078	0.172	0.284	0.417	0.572
∆P (mbar)	1.654	3.497	5.487	7.545	9.55	0.232	0.463	0.695	0.926	1.157
ANN Output	Mean change percentage in T					Mean change percentage in φ				
	2	4	6	8	10	2	4	6	8	10
Re	0.212	0.613	1.297	2.015	2.447	0.027	0.057	0.091	0.129	0.171
∆P (mbar)	0.71	1.43	2.19	3.11	4.07	0.062	0.124	0.186	0.248	0.310

The sensitivity analysis at FR = 8 LPM, summarized in Table 4, indicates that T variations exerted a substantially stronger influence on the ANN-predicted responses than equivalent perturbations in φ. For Re prediction, increasing the T perturbation from 2% to 10% increased the maximum response variation from 2.232% to 12.162%, whereas the corresponding φ-induced variation increased from only 0.272% to 2.405%. A similar trend was observed for the mean response levels: the mean Re variation increased from 1.651% to 8.298% under T perturbations, whereas the mean φ variation increased from 0.122% to 0.834%. These results indicate that T-dependent variations increasingly influenced flow inertia as the FR increased, whereas the contribution of φ remained comparatively limited. Increasing T perturbation from 2% to 10% increased the maximum ∆P variation from 0.144% to 0.653%, while the corresponding φ-induced variation remained limited between 0.042% and 0.218%. Likewise, the mean ∆P variation increased from 0.065% to 0.329% under T perturbations, whereas φ variations generated comparatively smaller changes ranging from 0.023% to 0.120%. Compared with the Re prediction, the lower sensitivity magnitudes observed for ∆P indicated a smoother dependence of pressure loss on the investigated operating parameters under elevated FR conditions.

**Table 4:** Sensitivity of ANN-predicted Re and ∆P to variations in φ and T at 8 LPM.

ANN Output	Max change percentage in T					Max change percentage in φ				
	2	4	6	8	10	2	4	6	8	10
Re	2.232	4.745	7.378	9.915	12.162	0.272	0.679	1.195	1.783	2.405
∆P (mbar)	0.144	0.286	0.421	0.544	0.653	0.042	0.086	0.13	0.174	0.218
ANN Output	Mean change percentage in T					Mean change percentage in φ				
	2	4	6	8	10	2	4	6	8	10
Re	1.651	3.326	5.01	6.676	8.298	0.122	0.272	0.446	0.636	0.834
∆P (mbar)	0.065	0.130	0.196	0.263	0.329	0.023	0.047	0.071	0.096	0.120

The sensitivity analysis at FR = 11 LPM, summarized in Table 5, reveals a further increase in the influence of T variations on ANN-predicted responses, particularly for Re prediction. Increasing T perturbation from 2% to 10% increased the maximum Re variation from 3.205% to 15.127%, whereas the corresponding φ-induced variation remained substantially lower, varying between 1.064% and 1.725%. A similar trend was observed for the mean response levels: the mean Re variation increased from 2.049% to 6.145% under T perturbations, whereas φ variations produced comparatively smaller changes, ranging from 0.179% to 0.443%. These results indicate that T-dependent variations increasingly dominated Re behavior under highly inertia-dominated flow conditions, whereas the contribution of φ remained secondary throughout the investigated perturbation range. For ∆P prediction, the sensitivity magnitudes remained considerably lower than those obtained for Re. Increasing T perturbation from 2% to 10% increased the maximum ∆P variation from 0.109% to 0.384%, while the corresponding φ-induced variation ranged from 0.095% to 0.529%. Similarly, the mean ∆P variation increased from 0.059% to 0.265% under T perturbations, whereas φ perturbations produced smaller changes between 0.023% and 0.120%. Compared with the Re prediction, the reduced sensitivity magnitude observed for ∆P indicated a smoother dependence of pressure-loss behavior on the investigated operating parameters, even at elevated FR conditions.

**Table 5:** Sensitivity of ANN-predicted Re and ∆P to variations in φ and T at 11 LPM.

ANN Output	Max change percentage in T					Max change percentage in φ				
	2	4	6	8	10	2	4	6	8	10
Re	3.205	6.478	8.569	11.865	15.127	1.064	1.609	1.623	1.099	0.7252
∆P (mbar)	0.109	0.202	0.278	0.338	0.384	0.095	0.193	0.296	0.408	0.529
ANN Output	Mean change percentage in T					Mean change percentage in φ				
	2	4	6	8	10	2	4	6	8	10
Re	2.049	3.557	5.323	5.609	6.145	0.259	0.407	0.443	0.3663	0.179
∆P (mbar)	0.059	0.112	0.166	0.217	0.265	0.023	0.047	0.071	0.096	0.120

## Conclusion

This study examined the capability of an ANN to represent the relationship between φ and T and the resulting flow characteristics, namely Re and ∆P, under fixed flow-rate conditions. The modeling framework was designed to resolve the inherently nonlinear and coupled thermo-compositional effects governing flow inertia and ∆P behavior, without imposing predefined functional correlations. To ensure statistical rigor and reliable predictive performance, the model’s predictive performance was evaluated through a comprehensive validation strategy that integrated cross-validation, performance-function monitoring, regression consistency analysis, error characterization, and sensitivity assessment to input perturbations. This approach enabled a systematic examination of model reliability, generalization capability, and predictive consistency across different flow regimes. The findings of this study are:

- For the Re at 5 LPM, 5-fold cross-validation yielded a mean MSE of 46.32 and a mean RMSE of 6.39, with fold-wise RMSE values ranging from 3.30 to 9.56, indicating statistically bounded variability and stable predictive behavior under low-inertia flow conditions.
- For the ∆P at 5 LPM, the corresponding mean MSE and RMSE were 8.51 mbar² and 2.68 mbar, respectively, while the RMSE range of 1.41–4.50 mbar confirmed consistent generalization across validation folds at a fixed FR.
- At 8 LPM, the Re prediction attains a mean MSE of 42.01 and a mean RMSE of 6.16, with RMSE values ranging from 3.94 to 9.59, demonstrating maintained predictive stability despite increased inertial sensitivity.
- For the ∆P at 8 LPM, cross-validation resulted in a mean MSE of 0.37 mbar² and a mean RMSE of 0.56 mbar, with RMSE confined to 0.29–0.98 mbar, indicating high predictive fidelity in the moderate-to-high flow regime.
- At the highest investigated FR of 11 LPM, Re prediction showed a mean MSE of 205.59 and a mean RMSE of 13.87, with RMSE ranging from 9.12 to 18.59, reflecting the increased complexity and sensitivity characteristic of inertia-dominated flows.
- For the ∆P at 11 LPM, the mean MSE and RMSE were 0.95 mbar² and 0.94 mbar, respectively, with RMSE values between 0.71 and 1.27 mbar, indicating that prediction errors remained bounded even under strongly inertia-dominated conditions.
- For the Re at 5 LPM, the error analysis yielded an overall MSE of 7.41, with training, validation, and test MSEs of 1.07, 7.84, and 26.00, respectively, indicating accurate learning of dominant flow characteristics alongside increased complexity in independent test conditions.
- For the ∆P at 5 LPM, the model achieved an overall MSE of 0.32 mbar², together with training, validation, and test MSEs of 0.027, 0.33, and 1.17 mbar², demonstrating stable learning and consistent representation of ∆P mechanisms.
- At FR = 8 LPM, the Re prediction showed an overall MSE of 7.07, with training, validation, and test MSEs of 4.22, 13.70, and 8.98, respectively. The close agreement between validation and test errors confirmed robust generalization without evidence of systematic bias.
- For the ∆P at 8 LPM, the corresponding performance metrics included an overall MSE of 0.1067 mbar², with training, validation, and test MSEs of 0.0276, 0.0999, and 0.3508 mbar², indicating reliable predictive behavior as flow inertia increased.
- At 11 LPM, the Re performance showed an overall MSE of 42.40, along with training, validation, and test MSEs of 28.39, 86.20, and 40.62, reflecting the increased complexity and sensitivity associated with inertia-dominated flow conditions.
- For the ∆P at 11 LPM, the model attained an overall MSE of 0.0565 mbar², with training, validation, and test MSEs of 0.0111, 0.0850, and 0.1643 mbar², respectively, confirming stable, well-bounded predictive behavior even at the highest investigated FR.
- Regression analysis demonstrated a strong linear relationship for Re, with overall R values of 0.99806 at 5 LPM, 0.99743 at 8 LPM, and 0.96796 at 11 LPM, indicating a high level of correlation, with only a modest reduction at the highest FR.
- For the ∆P, regression analysis similarly confirmed strong linear agreement, yielding R values of 0.99653 at 5 LPM, 0.97702 at 8 LPM, and 0.99419 at 11 LPM, indicating consistently strong predictive agreement across the investigated FRs.
- Relative error analysis for Re showed well-bounded prediction errors, with minimum–maximum ranges of 0.85–3.21% at 5 LPM, 0.35–7.42% at 8 LPM, and 0.15–4.48% at 11 LPM, indicating generally high predictive fidelity over varying flow regimes.
- For ∆P, relative errors remained tightly constrained, with minimum–maximum ranges of 0.22–3.18% at 5 LPM, 0.28–1.92% at 8 LPM, and 0.19–1.33% at 11 LPM, demonstrating stable prediction of ∆P behavior.
- Relative error analysis for Re confirms that prediction deviations remained well controlled across all investigated FRs, with mean relative errors of 4.64% at 5 LPM, 0.51% at 8 LPM, and 1.29% at 11 LPM, reflecting stable predictive performance despite increasing inertial effects at higher FRs.
- For ∆P, relative error analysis showed consistently low normalized prediction errors, with mean relative errors of 0.917% at 5 LPM, 0.769% at 8 LPM, and 1.252% at 11 LPM, indicating reliable modeling of ∆P behavior across the full range of operating conditions.
- Sensitivity analysis identified T as the dominant source of uncertainty for both Re and ∆P across all FRs, while the influence of φ remains consistently secondary.
- The ∆P showed markedly lower sensitivity to input perturbations than the Re, indicating a smoother, less nonlinear dependence on the governing parameters within the investigated operating range.

Although the developed ANN framework demonstrated stable predictive capability under the investigated conditions, the present model was established and validated using a specific PFHE geometry, a single NF configuration, and a limited set of operating conditions corresponding to three FRs. Consequently, the applicability of the current model should be interpreted within the investigated parameter space. Extending the proposed framework to broader operating conditions, alternative heat-exchanger geometries, and different nanofluid systems requires additional datasets and further validation studies. Furthermore, predictive uncertainty may increase outside the investigated training range due to measurement noise, thermophysical-property variability, and nonlinear hydrodynamic effects that are not fully represented in the available experimental dataset.

## Data Availability

The data that support the findings of this study are available from the corresponding author, upon reasonable request.

## Abbreviations

ANN: Artificial neural network
RMSE: Root mean squared error
MSE: Mean squared error
R: Correlation coefficients
LM: Levenberg–marquardt
PFHEs: Plate-fin heat exchangers
Re: Reynolds number
∆P: Pressure drop
FR: Flow rate
T: Temperature
NP: Nanoparticle
φ: Volume fraction
